# Reprogramming of Cell Fate During Root Regeneration by Transcriptional and Epigenetic Networks

**DOI:** 10.3389/fpls.2020.00317

**Published:** 2020-03-25

**Authors:** Tingting Jing, Rhomi Ardiansyah, Qijiang Xu, Qian Xing, Ralf Müller-Xing

**Affiliations:** ^1^Key Laboratory of Saline-Alkali Vegetation Ecology Restoration (Northeast Forestry University), Ministry of Education, Harbin, China; ^2^Institute of Development, College of Life Science, Northeast Forestry University, Harbin, China; ^3^Institute of Genetics, College of Life Science, Northeast Forestry University, Harbin, China

**Keywords:** root regeneration, adventitious roots, DNRR, callus, pluripotency, transcriptional networks, epigenetics

## Abstract

Many plant species are able to regenerate adventitious roots either directly from aerial organs such as leaves or stems, in particularly after detachment (cutting), or indirectly, from over-proliferating tissue termed callus. In agriculture, this capacity of *de novo* root formation from cuttings can be used to clonally propagate several important crop plants including cassava, potato, sugar cane, banana and various fruit or timber trees. Direct and indirect *de novo* root regeneration (DNRR) originates from pluripotent cells of the pericycle tissue, from other root-competent cells or from non-root-competent cells that first dedifferentiate. Independently of their origin, the cells convert into root founder cells, which go through proliferation and differentiation subsequently forming functional root meristems, root primordia and the complete root. Recent studies in the model plants *Arabidopsis thaliana* and rice have identified several key regulators building in response to the phytohormone auxin transcriptional networks that are involved in both callus formation and DNRR. In both cases, epigenetic regulation seems essential for the dynamic reprogramming of cell fate, which is correlated with local and global changes of the chromatin states that might ensure the correct spatiotemporal expression pattern of the key regulators. Future approaches might investigate in greater detail whether and how the transcriptional key regulators and the writers, erasers, and readers of epigenetic modifications interact to control DNRR.

## Introduction

During embryogenesis of higher plants, the shoot apical meristem (SAM) and the root apical meristem (RAM) are established at the opposite poles of the central axis. It is assumed that all other meristems derive from these two types of embryonic meristems, although all secondary SAMs or RAMs arise from partially differentiated cells, which need to go through reprogramming to become meristematic cells again. Root branching is based on these *de novo* RAMs that give rise to extensive root networks enabling plants to gain a stable hold in the ground, explore the soil and facilitate the uptake of water and mineral nutrients (Casimiro et al., 2003; Gonzali et al., 2005). Although the root system is genetically determined, they display a high plasticity in response to environmental variables such as water availability, nutrient levels, physical barriers or damage (Al-Ghazi et al., 2003; Sena and Birnbaum, 2010; Sugimoto et al., 2010; van Norman et al., 2013).

In dicotyledonous plants, such as *Arabidopsis thaliana* (*Arabidopsis*), the primary root grows as a thick central taproot (Bellini et al., 2014). Lateral roots (LRs) emerge post-embryonically and derive from pericycle cells close to the xylem pole cells of the primary root (De Smet et al., 2006); nonetheless, the morphology of primary root and LRs is basically identical (Birnbaum, 2016). Furthermore, adventitious roots (ARs) can be formed directly from various aerial organs (Fattorini et al., 2018) or indirectly from callus (Verstraeten et al., 2014). Depending on the status of the AR source cells, they can be directly fate-converted to AR root founder cells by a root-inducing signal or they first have to acquire root competence involving dedifferentiation (Druege et al., 2019). The natural ability of plants to regenerate is widely used in tissue culture, modern horticulture, and agriculture (Sussex, 2008; Druege and Franken, 2019). In rice and other cereals, ARs represent the main components of the root system as the primary root originating from the embryonic RAM is short-lived. During post-embryonic development, shoot-borne ARs form from nodes of the stem (Kawata et al., 1963; Liu et al., 2005). Some researchers prefer the term shoot-borne crown roots for these type of ARs, because they are part of the normal developmental program of cereals (Hochholdinger et al., 2004; Zhao et al., 2009). Nevertheless, ARs are also a common part of the regular root system of *Arabidopsis* under natural growth conditions in soil (Sheng et al., 2017). In recent years, research on plant root systems has made significant progress not only on natural root development but also on *de novo* root regeneration (DNRR). Besides classical DNRR research, which deals with the origin of ARs, some root regeneration studies have focused on reestablishment of the main RAM after pruning the root meristem tip (Efroni et al., 2016) or the replacement of single cells (Marhava et al., 2019). Here, we will give an overview of root regeneration systems and describe how recent breakthroughs in the model plants *Arabidopsis* and other plant species have changed our view of the molecular basis of cell fate reprogramming during DNRR focusing on transcriptional and epigenetic gene regulation. Furthermore, we will provide a short summary of the role of phytohormone signaling in DNRR, but the reviews of Lakehal and Bellini (2019) and Druege et al. (2019) provide a more comprehensive view on hormonal crosstalk and hormone-metabolic interactions in excision-induced AR formation.

### The Role of Auxin in DNRR Systems

Adventitious shoots and roots, derived from isolated or injured tissues and organs, provide an important survival strategy for plants in natural conditions (Duclercq et al., 2011; Sugimoto et al., 2011; Chen et al., 2014). In 1957, Skoog and Miller made the breakthrough discovery of experimentally induced phytohormone-dependent *de novo* regeneration of shoot and roots (Skoog and Miller, 1957). This gave rise to tissue culture methods which are still in use today in agriculture, industry and research (Duclercq et al., 2011). Hence, we can distinguish two types of *de novo* shoot and root regeneration, one under tissue culture conditions and the other in natural surrounding (Chen et al., 2014; Yu et al., 2017). In tissue culture, isolated plant tissues or organs named explants are cultured on nutrient-rich media containing an appropriate ratio of the phytohormones auxin and cytokinin, which can promote root or shoot formation in a controlled manner (Skoog and Miller, 1957). Under natural conditions, isolated organs can produce adventitious shoots and roots, and in some cases form whole plants: for example, some species from the Crassulaceae family are able to regenerate shoots and roots from leaves placed on soil (Chen et al., 2014; Xu and Huang, 2014; Ikeuchi et al., 2016). Nevertheless, endogenous hormones are crucial to induce adventitious shoot and root formation under natural conditions, for example in petunia, polar auxin transport and early IAA accumulation are essential for AR formation (Ahkami et al., 2013; Xu and Huang, 2014).

Auxin plays an important role in root growth and development, especially in LR and AR initiation (Klerk et al., 1999; Overvoorde et al., 2010; Lavenus et al., 2013; Bellini et al., 2014). ARs initiate near the wounding site of detached organs, which likely depends on auxin accumulation in the area (Liu et al., 2014). ARs can form from young *Arabidopsis* leaves without application of exogenous auxin, whereas exogenous auxin can increase the chance of root regeneration from older leaves that have decreased levels of endogenous auxin (Shoji et al., 1951; Chen et al., 2014). In addition, DNRR from most trees and other hard-to-root plants requires the application of exogenous auxin to induce ARs (Klerk et al., 1999; Díaz-Sala, 2014). The earliest studies on DNRR were carried out by Zimmerman and Hitchcock (1935) using aerial roots of grapes (*Vitis* sp.). These did not develop LRs until touching the soil, however, several new roots occurred if the aerial roots were cut and placed in solutions containing “root-forming” substances (Zimmerman and Hitchcock, 1935). In the same year several growth substances were tested for their ability to promote ARs and alpha-naphthaleneacetic acid (NAA) and indolebutyric acid (IBA) were found to be the most effective root-forming substances (Zimmerman and Wilcoxon, 1935). Exogenous application of the natural auxin indole-acetic acid (IAA) can induce a large number of roots from tomato leaf explants (Coleman et al., 1980). *In vitro*, a supply of exogenous sucrose also supports root regeneration by providing carbohydrate for plant growth (Calamar and de Klerk, 2002) and leaf explants fail to induce roots when growing on media in the dark without sucrose (Chen et al., 2014). A low concentration of sugar promotes LR initiation, while a high concentration of sugar inhibits LR initiation (Malamy and Ryan, 2001).

**FIGURE 1:**
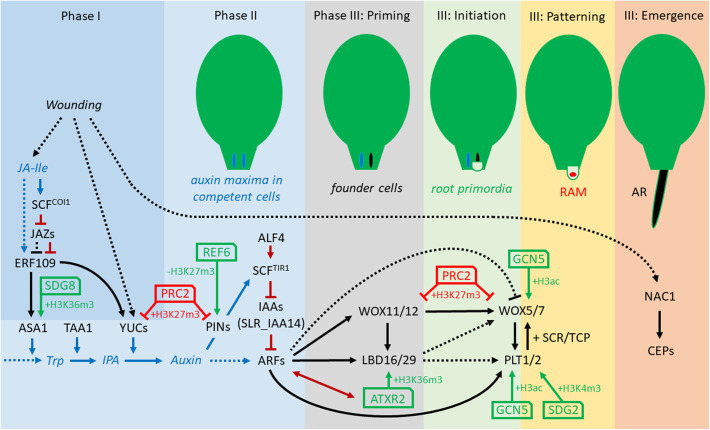
Concept of transcriptional and epigenetic regulation network during direct excision-induced DNRR in *Arabidopsis*. The three phases and four stages of phase III, priming, initiation, pattering, and emergence, are according to Yu et al. (2017) and Xu (2018). Note that several relations are not yet verified for DNRR but demonstrated for callus formation, LR initiation and/or regular root development. Black arrows, direct transcriptional regulation; dotted arrows, indirect regulation; blue arrows, JA and auxin synthesis and transport; dark-red arrows, protein-protein interaction; green arrows, positive epigenetic regulation; red arrows, negative epigenetic regulation. ALF4, ABERRANT LATERAL ROOT FORMATION4; ARFs, AUXIN RESPONSE FACTORs; ASA1, ANTHRANILATE SYNTHASE α1; ATXR2, ARABIDOPSIS THALIANA TRITHORAX-RELATED2; CEP, Cys endopeptidase; ERF109, ETHYLENE RESPONSE FACTOR109; GCN5, GENERAL CONTROL NON-REPRESSED5; H3K27me3, histone H3 tri-methylation of Lys27; IAAs, INDOLE-3-ACETIC ACID INDUCIBLEs; IPA, indole-3-pyruvic acid; JA-Ile, jasmonoyl-isoleucine; LBD16/29, LATERAL ORGAN BOUNDARIES DOMAIN16/29; NAC1, NAM/ATAF/CUC domain1; PINs, PIN-FORMED proteins; PLT1/2, PLETHORA1/2; PRC2, Polycomb Repressive Complex2; REF6, RELATIVE OF EARLY FLOWERING6; SDG2, SET-DOMAIN GROUP2; SLR_IAA14, SOLITARY ROOT_ INDOLE-3-ACETIC ACID INDUCIBLE 14; TAA1, TRYPTOPHAN AMINOTRANSFERASE OF ARABIDOPSIS1; Trp, Tryptophan; WOX11/12, WUSCHEL-RELATED HOMEOBOX11/12; WOX5/7, WUSCHEL-RELATED HOMEOBOX5/7; YUCs, YUCCAs.

In tissue culture, the ratio of auxin to cytokinin can influence *de novo* organogenesis in nutrient-rich growth media. Callus-inducing medium (CIM) has a balanced ratio of auxin to cytokinin. The transfer of the callus to root-inducing medium (RIM) with a high ratio of auxin-to-cytokinin induces root regeneration, whereas transfer to media with a low ratio induces shoot regeneration (Skoog and Miller, 1957; Valvekens et al., 1988). Recently, it was shown that the transfer from CIM with high auxin levels to B5 medium without auxin triggers AR formation as well (Yu et al., 2017). Callus, which derives from pericycle or related cells in the vascular tissue, depends on several key transcriptional regulators, which are also involved in LR and AR formation (Che et al., 2007; Atta et al., 2009; Sugimoto et al., 2010; Kareem et al., 2015; Ikeuchi et al., 2016). Root regeneration from callus might be one of the simplest case of cell fate reprogramming in plants since callus itself has an identity resembling that of lateral root primordia (LRP) (Sugimoto et al., 2010). Interestingly, the induction of so-called endogenous callus, which is presumed to be the first required step for DNRR from leaf explants, can be achieved without the application of exogenous auxin (Bustillo-Avendaño et al., 2018). Recently, AR formation from *Arabidopsis* leaf explants was intensively studied and can be divided into three phases: (I) early after wounding, signaling pathways trigger (II) auxin production in so-called converter cells (0–4 hours after leaf explant detachment [HAD]), followed by auxin accumulation in the region of AR formation by directed auxin transport (around 12 HAD) and (III) fate transition from regeneration-competent cells into fully formed ARs (Xu, 2018). The fate transition phase can be subdivided into four steps: During the “priming” step (24–48 HAD), regeneration-competent cells became root founder cells by cell fate transition; in the “initiation” step, root founder cells start to divide to form a dome-shaped LRP (48–96 HAD); during the “patterning” step, continuous cell division and differentiation generate a well-organized RAM, whereas in the “emergence” step the new formed AR breaks through the epidermis of the leaf explant (Figure 1; Yu et al., 2017; Xu, 2018).

Root regeneration from detached organs relies on neo-biosynthesis of endogenous auxin, which is partly induced by wounding and/or the auxin accumulation resulting from the cutting off of the basal auxin drain (Cai et al., 2014; Liu et al., 2014; Chen X. et al., 2016; Druege et al., 2019; Zhang et al., 2019). Near the wounding sites, auxin level can increase rapidly in mesophyll cells, and then polar transport results in auxin accumulating in competent cells of procambium and vascular parenchyma to trigger cell fate transition (Liu et al., 2014; Chen L. et al., 2016). Loss of function of auxin influx carriers (AUX1 and LAX3) and auxin efflux carriers (ABCB19) reduces the regenerative potential of hypocotyl and leaf explants, demonstrating the importance of auxin transport for DNRR (Sukumar et al., 2013; Della Rovere et al., 2015; Bustillo-Avendaño et al., 2018). One of the main endogenous auxin biosynthesis pathways is conducted in two steps (Figure 1): first, the TRYPTOPHAN AMINOTRANSFERASE OF ARABIDOPSIS (TAA) family of aminotransferases converts the main precursor for IAA, tryptophan (Trp) to indole-3-pyruvic acid (IPA). Then, the YUCCA (YUC) family of flavin monooxygenases participate in the conversion of IPA to IAA (Mashiguchi et al., 2011; Won et al., 2011). During DNRR, the expression of *YUC1* and *YUC4* is up-regulated in response to wounding, which promotes auxin production in both mesophyll cells and competent cells resulting in cell fate transition (Table 1; Chen L. et al., 2016). The *TAA1* mutation *weak ethylene insensitive 8-1 (wei8-1)* causes defects in AR formation, whereas the double mutant of *wei8-1 tar2-2* (*tryptophan aminotransferase related 2-2*) was mostly unable to regenerate AR from leaf explants (Sun et al., 2016). Similarly, double mutations in *YUC1*/*YUC4* and *YUC2*/*YUC6* can partially block the rooting of leaf explants, while in *yuc1246* quadruple mutants, rooting was severely blocked (Chen L. et al., 2016). In rice, *OsYUC1* overexpression causes massive proliferation of ARs or crown roots, respectively, whereas loss of *OsTAA1* reduces AR development (Zhang et al., 2018) confirming the central role of TAA and YUC mediated auxin biogenesis for AR formation that seems conserved between monocots and dicots.

**TABLE 1:** Selection of transcription factors and components of phytohormone signaling evidently or putatively involved in DNRR further indicating their role in primary and lateral root development or callus formation.

				**DNRR phases and stages**								
**Genes**	**PR**	**LR**	**Callus**	**I**	**II**	**III:**	**Pr**	**In**	**Pa**	**Em**	**Pro F**	**Comments**
*ABCB19 (MDR1)*	∙	∙**Δ**			**Δ**						IAA Tra	LFM display shorter LRs and reduced DNRR from hypocotyl explants, but DNRR is not impaired from intact hypocotyls (Wu et al., 2007; Sukumar et al., 2013)
*ALF4*	∙	∙**Δ**	**Δ**			**Δ**	**Δ**				IAA Sig	Regulator of SCF-TIR1 receptor, LFM acumulate IAAs, Exp in PR and LRs (Bagchi et al., 2018); LFM fail to produce LRs, callus and DNRR (Celenza et al., 1995; Sugimoto et al., 2010; Liu et al., 2014; Bustillo-Avendaño et al., 2018)
*ARF7,19*	∙	∙**Δ**	**Δ**	nd	nd	nd					IAA Sig TFs	Directly activate *LBD16/29* (Okushima et al., 2007), *ARF7* LFM have less LRs, double mutants fail to produce any LRs, whereas DNRR is normal but callus formation is reduced, Exp in the root vasculature, LRPs and developing LRs (Okushima et al., 2005; 2007; Liu et al., 2014; Lee et al., 2017)
*ASA1*	∙nd	∙ne**Δ**			**Δ**						IAA Syn	LFM have less DNRR (Zhang et al., 2019), less LRs in response to JA (Sun et al., 2009); Directly activated by ERF109 (Cai et al., 2014; Zhang et al., 2019)
*AUX1, LAX3*	∙	∙**Δ**			∙**Δ**						IAA Tra	LFM have less LRs and detached mutant hypocotyls show a reduced rooting capacity for ARs, Exp in PR, LRs and ARP (Marchant et al., 2002; Swarup et al., 2008; Della Rovere et al., 2013; 2015; Bustillo-Avendaño et al., 2018)
*COI1*	∙(**Δ**)	**Δ**		**Δ**							JA Sig	JA receptor, LFM causes reduced DNRR (Zhang et al., 2019) and LR formation in response to JA (Raya-González et al., 2012); Exp in RAM, LFM are insensitive to root growth inhibition by JA (Chen et al., 2011)
*ERF109*	nd	∙**Δ**		∙**Δ**							TF	JA induced after leaf detachment, LFM have reduced DNRR (Zhang et al., 2019); LFM have less LRs, ativates *ASA1* and *YUC2* (Cai et al., 2014)
*ERF115*	∙**Δ**		∙**Δ**	(**Δ)**		?			?		TF	Induced by JA, IAA and *ERF109* in protoxylem and QC cells, involved in root cell regeneration (Zhou et al., 2019); *ERF115-SRDX* blocks callus formation (Ikeuchi et al., 2017); controls QC cell devision (Heyman et al., 2013)
*FUS3, LEC2*	∙**Δ**	∙**Δ**			?						TFs	Two homologous B3 TFs interact to activate directly *YUC4* during LR formation (Tang et al., 2017); precocious growth of PR during embryogenesis (Vicente-Carbajosa and Carbonero, 2005)
*GA1 (CPS1), GA5 (GA20OX1)*						∙**Δ**					GA Syn	Ent-Copalyl Diphosphate Synthetase and GA 20-Oxidase, respectively; involved in vascular proliferation in DNRR, LFM have less AR capacity (Ibáñez et al., 2019)
*GAI*						∙**Δ**					GA Sig	*gai-1* is insensitive to GAs, involved in vascular proliferation during DNRR, LFM have reducted AR capacity (Ibáñez et al., 2019)
*IAA14 (SLR)*	∙(**Δ**)	**Δ**	**Δ**			**Δ**	**Δ**				IAA Sig TF	GFM, fail to produce LRs (Fukaki et al., 2002); less callus and DNRR (Shang et al., 2016; Bustillo-Avendaño et al., 2018); PR has less root hairs, Exp in RAM of PR and LRP (Fukaki et al., 2002; Vanneste et al., 2005)
*LBD16,29*	∙	∙**Δ**	**Δ**			∙**Δ**	∙**Δ**	(∙)			TFs	OE enhances AR formation (Liu et al., 2014); Direct target of ARF7/19 (Okushima et al., 2007); LFM have less LRs and callus (Fan et al., 2012)
*NAC1*		∙**Δ**				∙**Δ**				∙**Δ**	TF	Dominant-negative lines (*NAC1-SRDX*) have less LRs (Xie et al., 2000) and less ARs (Chen X. et al., 2016)
*PIN 1,2,3,7*	∙**Δ**	∙**Δ**			∙**Δ**						IAA Tra	Exp in leaf vasculature after excision, LFM have less ARs (Bustillo-Avendaño et al., 2018); Invoved PR and LR development (Petrásek and Friml, 2009)
*PLT1,2*	∙**Δ**	∙**Δ**				**Δ**		**Δ**			TFs	Activate *WOX5* (Shimotohno et al., 2018); double mutants: less DNRR (Bustillo-Avendaño et al., 2018), shorter PR but more LRs (Aida et al., 2004); Exp in LRPs and RAM of PR (Hofhuis et al., 2013; Du and Scheres, 2017)
*PLT3,5,7*		∙**Δ**	∙			?			?	?	TFs	Promotes LR emergence, triple mutants have less LRs, Exp in a subset of pericycle cells requiring ARF7/19 as activators (Hofhuis et al., 2013)
*SCR*	∙**Δ**	∙	∙			∙**Δ**		∙	∙	∙	TF	AR formation from hypocotyl is inhibited in LFM (Della Rovere et al., 2015); Involved in positioning the stem cell niche of RAMs, Exp in endodermis, QC and callus (Sabatini et al., 2003; Sugimoto et al., 2010; Kim et al., 2018)
*SHR*	∙**Δ**	∙**Δ**	∙			**Δ**		**Δ**			TF	LFM have reduced AR and LR formation as well as growth of the PR, Exp in the stelle, *shr plt1,2* triple mutants fails to produce ARs (Helariutta et al., 2000; Lucas et al., 2011; Della Rovere et al., 2015; Bustillo-Avendaño et al., 2018)
*TAA1 (WEI8), TAR2*	∙				∙**Δ**	∙					IAA Syn	Ubiquitously induced in leaf explants, double mutants are impaired in DNRR (Sun et al., 2016); Exp in RAM of PR (Stepanova et al., 2008)
*TCP20,21*	(∙**Δ**)					?			?		TFs	Interact with PLT1/3 and SCR to bind and induce *WOX5*, Exp in precursor QC cells (in embryos) (Shimotohno et al., 2018)
*WOX5,7*	∙**Δ**	∙**Δ**	∙nd			∙**Δ**		∙**Δ**	∙**Δ**	∙	TF	Activated by WOX11/12, *WOX5* LFM have reduced DNRR, which is enhanced in double mutants (Hu and Xu, 2016); *WOX5* maintains the stem cell niche of RAM, Exp in QC and callus (Sarkar et al., 2007; Sugimoto et al., 2010; Kim et al., 2018), whereas *WOX7* is involved in LR initiation (Kong et al., 2016)
*WOX11,12*			(**Δ**)			∙**Δ**	∙**Δ**				TF	LFM have less ARs, whereas OE inhibits AR and callus formation, Exp in AR founder cells, promotes *LBD16/19* (Liu et al., 2014; Sheng et al., 2017)
*YUC1,2,4,6*		∙**Δ**			∙**Δ**						IAA Syn	Induced in mesophyll cells of leaf explants, double mutants are partially, quadruple mutants severely impaired in DNRR (Chen L. et al., 2016), LFM of *YUC4* reduces LR formation (Tang et al., 2017)

*∙, expressed; ∆, mutant phenotype; ?, assumed involvement in DNRR; I, Phase I; II, Phase II; III:, Phase III:; Pr, Priming; In, Initiation; Pa, Patterning; En, Emergence; AR, adventitious root; ARP, adventitious root primordia; Exp, Expressed; GA sig, GA signaling; GA syn, GA biosynthesis; GFM, gain of function mutants; IAA sig, auxin signaling; IAA syn, auxin biosynthesis; IAA tra, auxin transport; JA sig, JA signaling; LFM, loss of function mutants; LR, lateral root formation; LRP, lateral root primordia; methylase, histone lysine methyl-transferase; nd, no defects observed; ne, not expressed; OE, overexpression; PR, primary (main) root; Pro F, protein function; QC, quiescent center; RAM, root apical meristem; TF(s), transcription factor(s).*

### Regulation of DNRR by Other Phytohormones

Beside the master player auxin, other phytohormones promote or antagonize DNRR: Cytokinin (CK), strigolactone (SL), and abscisic acid (ABA) suppress auxin production and/or AR formation whereas brassinosteroid (BR) and ethylene (ET) have positive effects (Table 1; Su and Zhang, 2014; Druege et al., 2019; Lakehal and Bellini, 2019). Although older studies suggest that gibberellin (GA) treatment inhibits AR formation (Busov et al., 2006; Mauriat et al., 2014), loss of components of GA synthesis (*GA1* and *GA5*, *GA REQUIRING*) or GA signaling (*GAI*, *GIBBERELLIC ACID INSENSITIVE*) causes defective vascular proliferation and consequently delayed AR formation in leaf explants indicating a positive role for GA in DNRR (Ibáñez et al., 2019). The stress phytohormone jasmonic acid (JA) and its biologically active form jasmonoyl-isoleucine (JA-Ile) are derivates of the lipid α-linolenic acid (Huang et al., 2017). After wounding, JA and JA-Ile levels increase rapidly in local and undamaged distal plant tissue (Glauser et al., 2008). Until recently, it was a matter of dispute whether JA facilitates or inhibits DNRR (Lakehal and Bellini, 2019). Ahkami et al. (2009) suggested that JA is a positive regulator of AR formation since it rapidly accumulates at the wounding site before ARs emerge in petunia leafy stem explants. On the other hand, continuous JA treatment inhibits AR formation whereas it promotes LR formation (Sun et al., 2009; Lischweski et al., 2015). The latter is partially inhibited in *yuc* mutants indicating that JA triggered LR formation is dependent on auxin synthesis (Sun et al., 2009; Cai et al., 2014). Reviewing the experimental details of diverse studies on the role of JA in adventitious rooting and considering relations to auxin homeostasis and signaling, Druege et al. (2019) recently provided a coherent explanation for the different findings and postulated that early, particularly wound-induced, JA accumulation stimulates AR formation in cuttings via IAA accumulation in the stem base and/or canalization toward AR source cells, while induction of invertases as molecular drivers of sink activity may be further involved. According to these theories, it was recently shown by Zhang et al. (2019) that in detached leaf explants, JA concentration and expression of JA response genes rise very quickly, reach a maxima after 1 hour and then decline in *Arabidopsis* leaf explants whereas auxin synthesis and other auxin-related genes reach their expression maxima one hour later (2 h after leaf detachment) indicating that JA signaling, triggered by wounding, precedes auxin signaling dependent cell fate reprogramming during DNRR. Similarly, ET biosynthesis is also triggered by wounding and stimulates AR formation at the stem base of cuttings in petunia, whereas auxin controls the timing of different phases of DNRR (Druege et al., 2014). There is indication in the literature that the ET signal is important for the early reprogramming of the AR source cells and may act via enhanced auxin level and/or sensitivity (Druege et al., 2019; Lakehal and Bellini, 2019).

### Key Transcriptional Regulators of DNRR

At the onset of DNRR in excised *Arabidopsis* leaves, wounding triggers the immediate accumulation of JA which activates the expression of *ERF109 (ETHYLENE RESPONSE FACTOR109)* encoding a transcription factor (TF) (Figure 1 and Table 1; Cai et al., 2014; Zhang et al., 2019). Loss-of-function mutants of *ERF109* or the JA receptor COI1 (CORONATINE INSENSITIVE1) display defective rooting from leaf explants (Zhang et al., 2019). ERF109 upregulates directly *ANTHRANILATE SYNTHASE*α*1* (*ASA1*) – a rate-limiting enzyme in tryptophan (Trp) biosynthesis – and *YUC2* indicating that ERF109 mediates cross-talk between JA and auxin biosynthesis during DNRR (Cai et al., 2014; Zhang et al., 2019). To prevent hypersensitivity to wounding, JASMONATE ZIM-domain (JAZ) repressors bind physically to ERF109 proteins and so inhibit the activation of *ASA1* by *ERF109* (Zhang et al., 2019). Later, the accumulation of auxin at the wounding site triggers the expression of further key TFs forming a regulatory network (Figure 1) that is initially different but subsequently identical to the transcriptional network controlling regular root development. Thereby, the signaling pathway from auxin perception to transcriptional responses consists of only two steps: First, the binding of auxin to the receptor and F-box protein TRANSPORT INHIBITOR RESISTANT1 (TIR1) triggers the ubiquitin-mediated degradation of the AUXIN/INDOLE-3-ACETIC ACID (Aux/IAA) transcriptional repressors. Then, the degradation of the Aux/IAAs breaks the physical inhibition of the AUXIN RESPONSE FACTORs (ARFs), which bind as transcriptional activators to auxin response elements (AuxREs) in the promoters of auxin response genes (reviewed in more detail in Weijers and Wagner, 2016). In *aberrant lateral root formation4-1* (*alf4-1*) mutants, the CULLIN1 subunit of the SCF^TIR1^ auxin receptor complex is destabilized leading to increases in the levels of Aux/IAA proteins, the repressors of ARFs (Bagchi et al., 2018). As the name implies, *alf4-1* mutant plants are impaired in lateral root formation (Celenza et al., 1995) but they also fail to regenerate roots from leaf explants (Liu et al., 2014). Furthermore, the *alf4-1* mutation blocks callus induction suggesting that callus and LR formation are under the same genetic control (Sugimoto et al., 2010). The reduction of auxin response in the *alf4* mutant during LR and callus formation is also caused by the increasing level of IAA14 (Perez-Garcia and Moreno-Risueno, 2018). *solitary root-1* (*slr-1*) mutants, which carry a dominant-negative version of IAA14, are not able to form regular LRs or callus on CIM (Shang et al., 2016) but can grow LRs after cutting the main root (root pruning) (Table 1; Xu et al., 2017).

During DNRR, the newly formed auxin maximum induces quickly the expression of *WUSCHEL-RELATED HOMEOBOX11* (*WOX11*) and its functional homolog *WOX12* in procambium and parenchyma cells, which mediates cell fate transition toward the establishment of root founder cells (Figure 1; Liu et al., 2014; Xu, 2018). Notably, the current state of research cannot rule-out that root founder cells of ARs can also initiate from differentiated cells beside procambium and parenchyma tissue via cell fate reprogramming (Yu et al., 2017). Although loss of *OsWOX11* causes severe growth defects including a near abolition of crown root production in rice, *wox11* and *wox12* single and double mutants show only slightly reduced AR formation in *Arabidopsis* (Zhao et al., 2009; Liu et al., 2014). The *WOX11* promoter region carries several AuxREs, which are essential for the auxin response indicating direct binding and activation of *WOX11* by ARFs (Liu et al., 2014). Although *WOX11* promotes AR and callus formations, *WOX11* is not involved in regular lateral root initiation (Liu et al., 2014; Sheng et al., 2017). The transition from root founder cells to root primordium cells is accompanied by decreasing *WOX11* and *WOX12* expression levels while those of *WOX5* and *WOX7* increase (Liu et al., 2014; Hu and Xu, 2016). This temporal succession of *WOX11* and *WOX5* expression is very similar to the temporal expression pattern during callus development supporting the idea that callus and AR initiation share the same genetic pathway at the cellular and molecular level (Liu et al., 2014). Recently, it has been shown that *WOX11/12* directly activate *WOX5/7* by binding to the promoters of *WOX5* and *WOX7* (Hu and Xu, 2016). *WOX5*, which encodes the functional homolog of the shoot stem cell promoting factor WUSCHEL (WUS), is expressed in the quiescent center (QC) of RAMs (Sarkar et al., 2007). Although *wox5-1* mutants form roots with disorganized RAMs, *wox5-1* mutant roots fail to maintain distal (columella) stem cells and, redundantly with the loss of other regulators, proximal stem cells during root development (Sarkar et al., 2007). In contrast to the activation of *WOX11/12* by ARFs, ARF10 and ARF16 repress and restrict *WOX5* to the QC (Ding and Friml, 2010). Interestingly, auxin is also required to activate *WOX5/7* expression in root founder cells, which divide to form root primordia cells during DNRR (Hu and Xu, 2016). As *WOX5* is expressed in callus as well as in RAMs of primary root, LRs and ARs, it is difficult to predict the stages of direct DNRR that would be affected by loss of *WOX5* (Table 1).

*WOX11/12* regulate at least partially the formation of AR and callus through activation of *LATERAL ORGAN BOUNDARIES DOMAIN16* (*LBD16*) and *LBD29* (Figure 1; Liu et al., 2014), whereby WOX11 directly binds to the WOX-binding sites in the *LBD16* promoter region (Sheng et al., 2017). The relationship between *WOX11* and *LBD16* is also important for shoot regeneration because both promote the pluripotency acquisition in callus cells (Liu et al., 2018). However, the activation of *LBD16* by WOX11 is not required for regular LRs that are also known as non-*WOX11*-mediated roots (Sheng et al., 2017). *LBD16*/*29* are also direct targets of *ARF7* and *ARF19* during lateral root formation, and *arf7-1 arf19-1* double mutants produce defective lateral roots (Okushima et al., 2005, 2007; Xu et al., 2017). Conversely, leaves of *arf7-1 arf19-1* double mutant are still able to induce root regeneration and callus formation (Liu et al., 2014; Lee et al., 2017) and can recover LRs after cutting the tip of the primary root (Sheng et al., 2017; Xu et al., 2017).

Although *LBD16* and *WOX5* are regulated by WOX11/12 and auxin, their expression patterns are different: *LBD16* is expressed in dividing root founder cells and the root primordia, but decreases during the formation of the root meristem whereas *WOX5* is restricted to the stem cell niche in the new RAM (Hu and Xu, 2016). Overexpression of *LBD16/29* can induce callus without exogenous auxin treatment, while LBD16-induced callus displays ectopic expression of *WOX5* and *PLETHORA* (*PLT1*) (Fan et al., 2012). *PLT1* and *PLT2* genes, whose transcription requires auxin accumulation and ARFs, are essential for QC specification and stem cell activity in the RAM (Aida et al., 2004). During embryogenesis and LR formation, PLT proteins physically interact with SCARECROW (SCR) and TCP (teosinte-branched cycloidea PCNA) TFs to specify and maintain the new formed QC and stem cell niche (Shimotohno et al., 2018). PLT–TCP–SCR complexes assemble on PLT-binding sites in the *WOX5* promoter to induce *WOX5* expression (Shimotohno et al., 2018). In turn, *WOX5* is needed for *PLT1* expression in RAMs (Ding and Friml, 2010). Interestingly, the *PLT* genes *PLT3/5/7*, which are essential in shoot regeneration, facilitate pluripotency in callus tissues by activating the root-specific stem cell regulators *PLT1* and *PLT2* (Kareem et al., 2015). During callus formation, *PLT1*/*2*, *WOX5*, and *SCR* can act as major regulators in the establishment and maintenance of cell regeneration capacity and possible pluripotency by inhibiting factors that in turn promote differentiation (Kim et al., 2018). *plt1 plt2* double mutants as well as *short-root* (*shr*) single mutants display significant reduction of leaf explants rooting and in rooting capacity (Bustillo-Avendaño et al., 2018). In *shr plt1 plt2* triple mutants, AR primordia initiation is fully blocked, because the postembryonic root founder cells cannot form. These results demonstrate the importance of *PLT1*, *PLT2*, and *SHR* for DNRR (Table 1; Bustillo-Avendaño et al., 2018).

During DNRR, the emergence of ARs seems an easy task but leaf explants expressing a dominant-negative version of the NAC1 TF (NAC1-SRDX) fail to grow-out ARs, although they are unaffected in the earlier steps of auxin-mediated cell fate transition (Chen X. et al., 2016). NAC1 induces the expression of *CEP* (*Cys endopeptidase*) genes, which encode proteins that might be involved in programed cell death and in degradation of extensin proteins in the cell wall (Chen X. et al., 2016). Therefore, the NAC1 pathway controls auxin-independently the emerging of ARs by loosening of cell walls of the surrounding tissue (Table 1).

Callus is an organized tissue similar to LRP (Sugimoto et al., 2010), while LRP and adventitious root primordia (ARP) differ only in their early steps of formation (Sheng et al., 2017). All three developmental events largely share TFs, which control the morphological changes in similar hierarchical networks (see model for DNRR in Figure 1). Most components of the transcriptional network are direct targets of ARFs that might suggest simultaneous expression rather than the observed activation in a chronological order. Obviously, the reciprocal and hierarchical regulations of the TFs contribute to their distinct spatiotemporal expression pattern. Nevertheless, epigenetic regulation can stabilize gene networks, restrict gene expression to specific tissue and/or provide a time buffer, which allows delayed transcriptional response to an upstream TFs (Müller and Goodrich, 2011; Xiao et al., 2016).

### Epigenetic Regulation of DNRR by the Repressive Mark H3K27me3

Epigenetic gene regulation here refers to mitotically or occasionally meiotically heritable changes in transcriptional activity that are not caused by changes in the DNA sequence but rather by covalent modifications to histone residues and DNA methylation (Bannister and Kouzarides, 2011; Smith and Meissner, 2013). The covalent nature of epigenetic chromatin modifications allows both stability through cell division as well as reversibility during development in response to extrinsic signals and endogenous clues (Bannister and Kouzarides, 2011; Müller-Xing et al., 2014b). For every chromatin modification like lysine methylation of histones exist both writers, which refers here to histone methyltransferases, and erasers, which refer here to histone demethylases, functioning as single proteins or protein complexes with enzyme activity (Xiao et al., 2016). Furthermore, chromatin modifications are specifically bound by so-called readers, proteins with domains which provide docking modules for the enzyme complexes (Andrews et al., 2016). While epigenetic regulation of callus formation and regular RAM development were subjects of several studies (Lee and Seo, 2018; Takatsuka and Umeda, 2015), the relationship of epigenetics and DNRR is a relatively new frontier, which was recently outlined as one of the hot topics for future research on adventitious rooting in cuttings (Chen L. et al., 2016; Lee et al., 2018; Druege et al., 2019; Zhang et al., 2019). To complete the current model of DNRR regulation and indicate future research directions, we also draw here parallels between the epigenetic control of callus formation, which is one of the earliest steps of cell fate reprogramming during AR initiation, and regular root development (Figure 1 and Table 2).

**TABLE 2:** Epigenetic factors evidently or putatively involved in DNRR further indicating their role in primary and lateral root development or callus formation.

				**DNRR phases and stages**							
**Genes**	**PR**	**LR**	**Callus**	**I**	**II**	**III:**	**Pr**	**In**	**Pa**	**Em**	**Comments**
*SDG2 (ATXR3)*	**Δ**	**Δ**	nd			?			?		H3K4me3 methylase involved in RAM organisation (Yao et al., 2013)
*ATX1*	**Δ**	**Δ**	nd			?					H3K4me3 methylase required for resrtriction of QC markers and for LR initiation, morphogenesis, and emergence (Napsucialy-Mendivil et al., 2014)
*SDG8*		**Δ**	nd	**Δ**							H3K36me3 methylase required for activation of *ASI1* by ERF109 (Zhang et al., 2019), LOF causes less LR (Cazzonelli et al., 2009)
*ATXR2*		**Δ**	**Δ**			**Δ**	**Δ**				H3K36me3 methylase, interacts with ARF7/9 and activates *LBD16/29* (Lee et al., 2017, 2018)
*CLF*	**Δ**	**Δ**	nd		?	?					H3K27me3 methylase of PRC2, represses PIN1, LOF causes increased RAM, root size and the number of LRs (Gu et al., 2014; Wang et al., 2019)
*SWN*	**Δ**		nd			?					H3K27me3 methylase of PRC2, LOF causes decreased RAM and root size (Lucas et al., 2016)
*CLF,SWN*	**Δ**	**Δ**	**Δ**		?	**Δ**					Double mutants fail to silence leaf identity genes, which prevent callus (He et al., 2012) and also AR formation (Liu et al., 2014)
*EMF2*	**Δ**	**Δ**	**Δ**		?	?					Component of PRC2, LOF inhibits PR growth, callus and LR formation (He et al., 2012; Gu et al., 2014)
*REF6*		**Δ**	nd			?	?				H3K27me3 demethylase, activates PIN1/3/7 that allows auxin transport and accumulation in LRP founder cells (Wang et al., 2019)
*PKL*	**Δ**					?					CHD3 chromatin remodeler, conteracts as TrxG factor PRC2 function and maintains root stem cells (Aichinger et al., 2009, 2011)
*HAG1 (GCN5)*	**Δ**		**Δ**!			nd					Histone acetyltransferase, activates PLT genes and maintains the stem cell niche (Kornet and Scheres, 2009), LOF causes faster callus growth (!) but does not impair indirect DNRR (Kim et al., 2018)

*∆, mutant phenotype; ?, assumed involvement in DNRR; I, Phase I; II, Phase II; III:, Phase III:; Pr, Priming; In, Initiation; Pa, Patterning; En, Emergence; AR, adventitious root; LOF, loss of function; LR, lateral root formation; LRP, lateral root primordia; methylase, histone lysine methyl-transferase; nd, no defects observed in callus formation (He et al.,2012) or indirect DNRR (Kim et al. 2018); PR, primary (main) root; RAM, root apical meristem; QC, quiescent center.*

Reprogramming of cell fate requires the activation and repression not only of a few genes but whole transcriptional networks controlling developmental programs and it is accompanied by local and global changes in epigenetic modifications (Lee and Seo, 2018). The Polycomb repressive complex 2 (PRC2) is a key “writer” which deposits the repressive marks di- and tri-methylation of Lys27 on histone H3 (H3K27me2/3). It is assembled from four highly conserved core components as well as a more variable collection of associated proteins, some of which are DNA binding proteins that may guide the PRC2 to Polycomb (PcG) target genes (Xiao et al., 2017; Zhou et al., 2018). In *Arabidopsis*, three partially redundant homologs *CURLY LEAF* (*CLF*), *MEDEA* (*MEA*), and *SWINGER* (*SWN*) encode the catalytic subunit of the PRC2 histone methyltransferase (HMT), while *EMBRYONIC FLOWER2* (*EMF2*) encodes a second core PRC2 component (Schmitges et al., 2011; Müller-Xing et al., 2014a). In contrast, RELATIVE OF EARLY FLOWERING6 (REF6) and its two close paralogs, EARLY FLOWERING6 and JMJ13, are three partially redundant H3K27me3 demethylases (erasers) with important functions in reprogramming during plant development (Table 2; Yan et al., 2018).

During callus formation from leaf explants, over 400 PcG targets lose H3K27me3, whereas less than 200 targets gain H3K27me3, indicating the significance of reactivation of former silenced genes (He et al., 2012). The levels of H3K27me3 decrease first at several genes of the auxin pathway including *YUC4*, *NITRILASE2* (*NIT2*), *IAA CARBOXYL METHYLTRANSFERASE1* (*IAMT1*), and *PIN-FORMED1* (*PIN1*). Subsequently, H3K27me3 levels increase at leaf specific genes but decrease at genes involved in root development suggesting a central role for the repressive epigenetic mark H3K27me3 during regeneration (He et al., 2012). *WOX11* expression, which is essential for establishing root founder cells during DNRR, depends on endogenic auxin synthesis by YUC proteins (Liu et al., 2014; Chen L. et al., 2016). The gene loci of *YUC1* and *YUC4*, as well as *WOX11*, are H3K27me3-marked PcG targets whose levels decline in callus tissue cultured on CIM (He et al., 2012; Liu et al., 2014; Chen L. et al., 2016). Principally, reduction of H3K27me3 levels at target genes can be achieved either by active removal through H3K27me3 demethylases or by H3K27me3 dilution through cell divisions (Sun et al., 2014). During DNRR from leaf explants, the activation of *YUC1* and *YUC4* expression is accompanied by decreasing H3K27me3 levels after only a few hours on B5 medium (Chen L. et al., 2016) suggesting an active removal, e.g., by H3K27me3 demethylases as little or no cell division and DNA replication occurs in this time period.

In primary root and LRs, the polar auxin transport and local auxin maxima are widely achieved by the family of PIN-FORMED (PIN) efflux carriers, which includes PIN1, PIN2, PIN3, PIN4, and PIN7 (Petrásek and Friml, 2009). PRC2 inhibits LR formation and root growth by depositing the repressive H3K27me3 mark on chromatin at the *PIN1* locus (Gu et al., 2014). Whereas the H3K27me3 demethylase REF6 binds to a specific DNA sequence (CTCTGYTY) and de-represses *PIN1/3/7* during LR formation (Lu et al., 2011; Cui et al., 2016; Wang et al., 2019). Accordingly, *ref6* mutants have fewer LRs than wild-type (Wang et al., 2019). In contrast, *ref6* mutants are not impaired in callus formation from leaf explants (He et al., 2012), the first step of AR initiation (Sheng et al., 2017). As the contribution of *REF6* to DNRR has not yet been tested, it remains unclear whether in *ref6* mutant explants, AR formation is unaffected, as suggested by the result of callus formation, or delayed like LR formation in *ref6*. During rice shoot development, OsWOX11 recruits the REF6 homolog JUMONJI705 (JMJ705) to promote gene expression by H3K27me3 demethylation (Cheng et al., 2018). It would be interesting to test whether WOX11 and REF6 cooperate similarly during regular root development and DNRR in *Arabidopsis*.

PRC2 complexes containing either the HMT CLF or SWN are essential for post-embryonic plant development (Gutzat et al., 2012) and apparently play a role in DNRR. Although *clf-50 swn-1* double mutants can form normal callus from root tissue, *clf-50 swn-1* double and *emf2* single mutants are defective in callus formation from leaf explants (He et al., 2012). In the latter case, the plants with reduced PRC2 activity fail to silence leaf identity genes such as *SAWTOOTH1* (*SAW1*) and *SAW2*, whereas the root identity genes *WOX5* and *SHR* are de-repressed as during normal callus formation (Kumar et al., 2007; He et al., 2012). Similarly, *clf-50 swn-1* double mutants fail to form AR from leaf explants (Liu et al., 2014) but it is not yet clear whether impaired silencing of *SAW1/2* or other leaf-regulatory genes plays a role. *swn clf* ± mutant explants, presumably harboring reductions in H3K27me3 levels, display an earlier re-activation of *WUS* enabling faster shoot regeneration (Zhang et al., 2017). This faster activation of *WUS* is likely achieved by easier access of the B-Type ARR and HD-ZIP class III TFs to the *WUS* chromatin (Zhang et al., 2017). Facilitated access to DNRR-related genes may also accelerate root regeneration in PcG mutants. Loss of *CLF* causes ectopic LR formation, a longer primary root (Gu et al., 2014), and higher root meristem activity as indicated by increased *WOX5* expression and meristem size (Aichinger et al., 2011). On the other hand, the loss of the *CLF* homolog *SWN* causes shorter roots and decrease in the RAM size indicating that root growth defects varies in different PcG mutants (Lucas et al., 2016). Hence, alternative CLF-PRC2 and SWN-PRC2 complexes might target selectively different genes, which either promote or inhibit regular root development, but possibly also DNRR (Table 2).

### Epigenetic Regulations of DNRR by Histone Modifications With Positive Effects on Transcription

Trithorax group (TrxG) proteins are a diverse group of antagonists of PcG-mediated gene repression that were originally defined genetically by their ability to suppress PcG mutant phenotypes (Kennison and Tamkun, 1992). Some TrxG proteins promote PcG target gene expression by catalyzing activation marks, others by removing the repressive H3K27me3 mark and yet others by chromatin remodeling to slide nucleosomes and facilitate access. The principle marks associated with transcriptional activation are di- and tri-methylation of histone H3 at lysine 4 and/or lysine 36 (H3K4me2/3 and H3K36me2/3) (Papp and Müller, 2006). In the early phase of DNRR, the upregulation of *ASA1* by JA-activated ERF109 required H3K36me3 pre-deposition by SET DOMAIN GROUP8 (SDG8), which is also involved in LR formation (Table 2; Cazzonelli et al., 2009; Zhang et al., 2019). Another H3K36me3 HMTase, ARABIDOPSIS THALIANA TRITHORAX-RELATED2 (ATXR2) is involved in callus and AR formation by activating *LBD16* and *LBD29* expression (Lee et al., 2017, 2018). ATXR2 binds directly to the promoter region of *LBD16* and *LBD29* to depositing H3K36me3 at these loci (Lee et al., 2017). In addition, ATXR2 interacts physically with the known activators of *LBD16* and *LBD29* ARF7 and ARF19, suggesting that these ARF TFs could recruit ATXR2 to both *LBD* promoters (Lee et al., 2017). *SDG2*, also known as ATXR3, encodes the main H3K4me3 HMTase (Berr et al., 2010; Guo et al., 2010) and is required for the organization and function of stem cell niche in the RAM (Yao et al., 2013). *sdg2-3* mutants have a shorter root and fewer LRs than wild type (Yao et al., 2013) but nonetheless are still able to induce callus formation (He et al., 2012), leaving the question unanswered whether DNRR initiation or only the later step of the emergence of ARs are impaired in *sdg2* mutants. The preliminary data concerning other TrxG proteins such as *ARABIDOPSIS THALIANA TRITHORAX1* (*ATX1*, encoding a H3K4me3 HMTase) are inconclusive or contradictory, for example inactivation causes reduced root length and LR number yet the mutants are able to form callus from leaf explant (He et al., 2012; Napsucialy-Mendivil et al., 2014). The chromatin remodeler PICKLE (PKL) counteract PcG function as *pkl* mutants display reduced expression of some PcG target genes (Aichinger et al., 2009) and have shorter roots and reduced root stem cell activity (Aichinger et al., 2011). It was proposed that PKL, together with PICKLE RELATED2 (PKR2), activate PcG targets outside of the RAM to promote cell differentiation, whereas PKL specifically maintains root stem cell (Aichinger et al., 2011). Plants carrying the *slr-1* mutation are blocked in auxin signaling, and so the initiation of LRs is blocked (Shang et al., 2016). In *slr-1 pkl* double mutants, LR formation is rescued in an ARF7/19 function dependent manner revealing that chromatin remodeling by PKL regulates negatively auxin-mediated LR formation (Fukaki et al., 2006). It remains to be tested whether PKL or PKR2 have a similar function in AR formation.

Histone lysine acetylation (Kac) may have more direct links to transcriptional control than most other chromatin modifications, as Kac promotes transcription not only by recruiting reader proteins but also by neutralizing the positive charge on the lysine side chain, which directly facilitates RNA synthesis by making the DNA more accessible to the transcriptional machinery (Zhao et al., 2018). Kac can overcome repressive histone marks such as H3K27me3 or can be replaced by such epigenetic marks. The histone acetyltransferase HISTONE ACETYLTRANSFERASE OF THE GNAT FAMILY1 (HAG1), also known as *A. thaliana* GENERAL CONTROL NON-REPRESSED5 (*At*GCN5) (Vlachonasios et al., 2003; Servet et al., 2010), plays a key role in the establishment of callus pluripotency and subsequent shoot regeneration (Kim et al., 2018). The expression of *PLT* genes is positively regulated by *At*GCN5 to maintain the stem cell niche in roots (Kornet and Scheres, 2009). *hag1-6* mutants display very short roots and a smaller root meristem size (Kornet and Scheres, 2009) but faster-growing callus on CIM, which is associated with decreasing expression of the root key regulators *WOX5*, *SCR*, *PLT1*, and *PLT2* (Kim et al., 2018). Although *hag1-6* mutant callus fails to induce shoot regeneration on shoot inducing medium (SIM), it is surprisingly able to regenerate roots after transferring from CIM to RIM (Table 2; Kim et al., 2018). In rice, OsWOX11 interacts with the ADA2-GCN5 histone acetyltransferase complex to activate downstream target genes during AR formation (Zhou et al., 2017) giving a textbook example of how TFs acts as recruiter of epigenetic factors (EFs) for long-term transcription regulation.

## Conclusion and Outlook

The integration of genes and their protein products in transcriptional networks, such as the one controlling DNRR, often start with the description of their mutant phenotypes and their expression patterns. Nevertheless, the components of transcriptional-epigenetic networks have to be interconnected through direct binding of one component to the promoter of another component or by direct protein-protein interaction or by phytohormone or other signaling pathways. These physical interactions need to be tested by yeast-two-hybrid (Y-2-H) or proteomics assays, chromatin immunoprecipitation (ChIP), electrophoretic mobility shift assay (EMSA), DNA-affinity precipitation assay (DAPA) and other approaches. Recent progress in DNRR research allowed us to develop a model on the transcriptional and epigenetic reprogramming network controlling DNRR (Figure 1). However, several links of the model are not yet verified in DNRR systems and several gaps remain obvious, for example: (1) Taking into account that *ERF109*, which functions upstream of *ERF115* (Zhou et al., 2019), is JA inducible, it would be interesting to test whether MYC2, the main JA response factor, can directly bind to the *ERF109* promoter or whether the loss of other JA signaling components impairs DNRR, like for mutations in the JA receptor COI1 (Zhang et al., 2019). (2) Although several publications showed that auxin can induce *NAC1* (Xie et al., 2000; Guo et al., 2005; Wang et al., 2006), a recent publication suggests that *NAC1* expression near the wounding side does not require auxin (Chen X. et al., 2016) raising the question whether JA or another signal could be the missing link. (3) Considering the linkages between ET signaling, the WIND (WOUND INDUCED DEDIFFERENTIATION) TF gene family, micro RNAs and epigenetic processes recently outlined by Druege et al. (2019), these relationships should additionally be taken into account. (4) It is also unclear whether WOX5/7 or PLT1/2 are direct targets of LBD16/29. Another open question is whether ARs only originates from cells, which are root regeneration-competent from the beginning or is it possible that ARs also derivate from cells, which have first to acquire root regeneration competence.

Writing and erasing of histone marks facilitates and stabilizes long-term changes in transcriptional programs. Therefore, the recruitment of EFs by long non-coding RNAs and TFs is highly important for DNRR yet our current knowledge is very limited and only a few interactions between TFs and EFs are known. Using DNRR-related TFs as bait, approaches, like Y-2-H screen or immunoaffinity-purification with subsequent mass-spectrometry, can identify further TF/EF protein complexes. On the other hand, if the up-stream TFs are unknown, they can be identified in yeast-one-hybrid screens using promoter sequences of DNRR-related genes.

DNRR itself is an inducible system that can give us temporal resolution of gene expression and changes of epigenetic marks during the reprogramming of cell fate. Nevertheless, most DNRR studies, involving ChIPs and other chromatin-related approaches, used callus, mixed callus/explant tissue or whole leaf explants. At least after accumulation of auxin in the competent cells, using the mixed tissue cannot provide the needed spatiotemporal resolution. An approach combining DNRR and INTACT (isolation of nuclei tagged in specific cell types) system (Deal and Henikoff, 2011) or classical protoplast sorting (Birnbaum et al., 2003) will provide more specific and accurate data.

The question of transferability of DNRR studies in *Arabidopsis* to crop plants and trees seems to be a big issue as *Arabidopsis* is still the main genetic and molecular biological tool in plant research. *Arabidopsis* belongs to the Brassicaceae family, which include many important agricultural crop varieties, such as canola and cabbage. Therefore, the knowledge gained from *Arabidopsis* research has also agricultural significance (Paulraj and Yeung, 2012). Interestingly, several key regulators of DNRR were first described in rice including (*Os*)*WOX11* and *ARL1*/*LOB29* (Liu et al., 2005; Zhao et al., 2009) with similar functions in AR development indicating a high degree of conservation of the DNRR transcriptional networks between monocots and dicots and therefore likely between most of the crop plants. Nevertheless, that has to be confirmed by further efforts to study the molecular mechanisms of DNRR in crops.

## Author Contributions

All authors listed have made a substantial, direct and intellectual contribution to the work, and approved it for publication.

## Conflict of Interest

The authors declare that the research was conducted in the absence of any commercial or financial relationships that could be construed as a potential conflict of interest.
