# Corrigendum: Reprogramming of Cell Fate During Root Regeneration by Transcriptional and Epigenetic Networks

**DOI:** 10.3389/fpls.2021.698412

**Published:** 2021-06-04

**Authors:** Tingting Jing, Rhomi Ardiansyah, Qijiang Xu, Qian Xing, Ralf Müller-Xing

**Affiliations:** ^1^Key Laboratory of Saline-Alkali Vegetation Ecology Restoration (Northeast Forestry University), Ministry of Education, Harbin, China; ^2^Institute of Development, College of Life Science, Northeast Forestry University, Harbin, China; ^3^Institute of Genetics, College of Life Science, Northeast Forestry University, Harbin, China

**Keywords:** root regeneration, adventitious roots, DNRR, callus, pluripotency, transcriptional networks, epigenetics

In the original article, **Tables 1** and **2** contained several errors as published. The errors are indicated below and the article has been updated.

In **Table 1**, of the 24 genes named, the following eight were spelled incorrectly:

- “*ABCB19* (*MDR1*)” was presented incorrectly as “*ABCB19* (*MDRlj*)”

- “*ASA1*” was presented incorrectly as “*ASM*”

- “*COI1*” was presented incorrectly as “*con*”

- “*GA1* (*CPS1*), *GA5* (*GA20OX1*)” was presented incorrectly as “*GA1* (*1*), *GA5* (*GA200X1*)”

- “*IAA14* (*SLR*)” was presented incorrectly as “*IAA14 SLR* (*IAA14*)”

- “*TAA1* (*WEI8*), *TAR2*” was presented incorrectly as “*TAA1* (*WEIS*), *TAR2*”

- “*WOX5,7*” was presented incorrectly as “*W0* × *5,7*”

- “*WOX11,12*” was presented incorrectly as “*W0* × *11,12*”

Several classifications of expression and/or mutant phenotypes were incorrect in **Table 1**.

In **Table 1**, the citation to reference “Du and Scheres (2018)” should have been to “**Du and Scheres (2017)**”; the reference in question was omitted from the original article and appears in the Reference section below.

**Table 1** had several minor typographical errors and omitted the explanations of the following abbreviations from the footnote: AR, adventitious root; ARP, adventitious root primordia; LRP, lateral root primordia; OE, overexpression; QC, quiescent center; RAM, root apical meristem.

Finally, the order of the genes in **Table 1** was incorrect, with “*AUX1, LAX3*” appearing out of sequence.

**Table 2** had several minor typographical errors and omitted the explanations of the following abbreviations from the footnote: AR, adventitious root; LRP, lateral root primordia; RAM, root apical meristem; QC, quiescent center.

The authors apologize for these errors and state that they do not change the scientific conclusions of the review article in any way. The original article has been updated.

## References

[B1] Du Y. Scheres B. (2017). PLETHORA transcription factors orchestrate *de novo* organ patterning during *Arabidopsis* lateral root outgrowth. Proc. Natl. Acad. Sci. U.S.A. 114, 11709–11714. 10.1073/pnas.1714410114 29078398 PMC5676933

